# Beyond the Numbers: How Clinical Performance Metrics Impact Emergency Medicine Residents

**DOI:** 10.5811/westjem.50814

**Published:** 2026-05-12

**Authors:** Catherine Burger, Matthew Pirotte, Kaitlin Ray, Joseph Sikon, Kendra Parekh

**Affiliations:** * Duke University School of Medicine, Department of Emergency Medicine, Durham, North Carolina; † Vanderbilt University Medical Center, Department of Emergency Medicine, Nashville, Tennessee

## Abstract

**Introduction:**

Emergency physicians commonly receive feedback in the form of performance metrics such as patients seen per hour. Reviewing metrics has been associated with increased stress and burnout. Although effects on efficiency have been examined, studies have not yet investigated the potential psychological and motivational impacts of providing performance metrics to residents during training. In this study we explore residents’ interest in receiving performance metrics during training and how receiving performance metrics might affect their 1) perceived pressure and motivation to change performance, 2) perspectives on the importance and actionability of metrics, 3) perceived readiness to receive metrics after graduation, and 4) possible effects on their postgraduate career plans.

**Methods:**

Senior emergency medicine residents at a single, quaternary-care training center completed an anonymous pre-metric survey using a 5-point Likert scale of agreement (1 = strongly agree, 5 = strongly disagree) on the psychological and motivational impacts of receiving performance metrics. All senior residents, regardless of survey completions, were then given the option to view their personal performance metrics in comparison to deidentified metrics for the senior classes. Residents who viewed their metrics were offered the opportunity to complete the post-metric survey, which was identical to the pre-metric survey. Resident interest in viewing their metrics was recorded, and we compared survey responses using unpaired *t*-tests.

**Results:**

All 26 residents (100%) chose to view their metrics, 25 (96%) completed the pre-metrics survey and 17 (73%) completed the post-metrics survey. After receiving performance metrics, residents reported feeling less pressure to change their performance (pre-metrics mean 2.32 [standard deviation 0.80]), post-metrics mean 3.05 [0.71], *P* < .01), and they reported feeling more prepared to receive metrics after graduation (pre-metrics mean 2.32 [0.95], post-metrics mean 1.68 [0.58], *P* = .01]. There was no significant change in residents’ responses to questions about metrics perceptions, motivation, or interest in administrative leadership after graduation.

**Conclusion:**

This single-site, academic study indicated that senior residents are interested in seeing their personalized and deidentified group performance metrics and that viewing these metrics increases their sense of preparedness for graduation without necessarily affecting the pressure or motivation they felt during training.

## INTRODUCTION

Within the field of emergency medicine (EM), clinicians, patients, and administrators all value efficient and timely care delivery.^1^–^4^ Some measures of care delivery include clinical productivity metrics, such as patients seen per hour and timeliness of documentation completion. To foster efficient and timely care, hospital leadership groups often provide physicians with individual performance metrics as well as national or local group-comparison metrics.^5^,^6^ Some EM residency programs also provide their residents with clinical productivity metrics during training. The goals of providing these metrics to trainees may include improving resident efficiency as well as educational preparation for their postgraduate careers.

Multiple studies have shown that providing EM residents productivity metrics is unlikely to change their efficiency during training.^7^,^8^ Recent research in EM has also shown that productivity metrics can be heavily influenced by factors outside the individual clinician’s control and that receiving feedback perceived as unactionable is distressing and contributes to burnout.^9^–^11^ This is in contrast to studies showing increased resident satisfaction with feedback when metrics were included.^8^ Few studies have looked at the potential motivational and psychological impacts of providing productivity metrics during EM training.

In this study we sought to explore, among senior EM residents, 1) whether there is interest in viewing their performance metrics and 2) whether viewing personal and de-identified group productivity metrics influences a) perceived pressure to change performance, b) motivation to change performance, and c) perceived preparedness to receive metrics after graduation. Secondary objectives included exploration of how receiving these metrics might affect residents’ perceptions of the importance and actionability of performance metrics. This information could help guide EM educators on how to best use productivity metrics during resident training.

## METHODS

This study, which was approved by the study site’s institutional review board, was conducted at a single quaternary-care teaching hospital with a three-year EM residency training program in the United States. In January 2025, all senior EM residents (postgraduate years 2 and 3) were invited to participate in two anonymous 6-question, online surveys via email using Research Electronic Data Capture (REDCap), hosted at Vanderbilt University Medical Center. The first survey was sent prior to residents viewing their performance metrics (pre-metrics survey), and the second was sent after they had the opportunity to view performance metrics (post-metrics survey). Both pre- and post-metric surveys were developed in accordance with survey design best practices.^12^ In the three years prior to this study, residents had not received any group or individual performance metrics. Participants had four days to complete the pre-metrics survey and were automatically sent reminders to complete the survey at 24, 48, and 72 hours after the initial survey invitation was sent.

The pre-metrics survey included two questions about the psychological impact of performance metrics, two questions about perspectives on performance metrics, one question about how prepared they felt to receive performance metrics after graduation, and one question about postgraduate career plans. Survey questions are listed in Table 1. The response scale was a 5-point Likert scale of agreement (1 = strongly agree, 5 = strongly disagree). After the initial 4-day survey period, the opportunity to view their productivity metrics was then offered to all senior residents, regardless of survey completion, with a clearly stated option to opt out of viewing their metrics. When offered the opportunity to view their metrics, residents were advised which metrics they would receive and that the metrics would not be included in their evaluations, regardless of their decision to view them or not. Residents were given one week to express their interest in viewing their metrics, and all who requested to view their metrics received an email with a copy of their metrics after the last day of the one-week request period.

**Population Health Research Capsule**

What do we already know about this issue?

*Emergency (EM) resident productivity does not significantly change when residents are given performance metrics*.

What was the research question?

*Do EM residents want to see their productivity metrics, and what motivational effects do these metrics have?*

What was the major finding of the study?

*Residents want to see their metrics, viewing decreased perceived pressure and increased perceived graduation preparedness*.

How does this improve population health?

*All 26 residents chose to see their metrics. Viewing decreased perceived pressure and increased perceived graduation preparedness*.

The clinical productivity metrics available to residents included the average number of patients seen per hour and percentage of charts completed within 24 hours, both of which were averaged over the preceding three months. Residents also received de-identified metrics for the PGY-2 and PGY-3 classes. We obtained the number of patients seen, number of hours worked, and chart completion within 24 hours from the electronic health record (EHR) at the primary clinical teaching site. Data from other sites were not included due to the use of different EHR systems. If a resident chose to view their metrics, they were then asked to complete the post-metrics survey after viewing their metrics. The pre- and post-metric surveys were identical. Participants had four days to complete the post-metrics survey and were automatically sent reminders to complete the survey at 24, 48, and 72 hours after the initial post-metrics survey invitation was sent. We recorded resident interest in viewing their metrics and analyzed survey results using descriptive statistics and unpaired *t*-tests to compare responses on the pre- and post-metrics surveys. Statistical analyses were performed using Prism GraphPad (Siemens Industry Software, Inc, Plano, TX).

## RESULTS

Of 26 residents, 25 (96%) completed the pre-metrics survey. All 26 residents (100%) requested to view their productivity metrics. Nineteen of 26 (73%) residents completed the post-metrics survey. The response options ranged from 1 (strongly agree) to 5 (strongly disagree). Pre- and post-metric responses can be found in Table 2. The mean (standard deviation) response for “receiving performance metrics makes me feel pressured to change my performance” was 2.32 (0.80) on the pre-metrics survey and 3.05 (0.71) on the post-metrics survey (*P* < .01). The mean response for “receiving performance metrics motivates me to change my performance” was 2.04 (0.68) on the pre-metrics survey and 2.11 (0.81) on the post-metrics survey (*P* = .77). The mean response for “performance metrics are an important measure of my work” was 1.88 (0.73) on the pre-metrics survey and 2.11 (0.81) on the post-metrics survey (*P* = .34). The mean response for “performance metrics are an actionable form of feedback (I am able to change my results)” was 1.96 (0.61) on the pre-metrics survey and 2.16 (0.60) on the post-metrics survey (*P* = .29). The mean response to “I feel prepared to receive emergency department performance metrics after graduation” was 2.32 (0.95) on the pre-metrics survey and 1.68 (0.58) on the post-metrics survey (*P* = .01). The mean response for “I am interested in exploring the operations and administration aspect of how an emergency department performs after graduation” was 1.92 (0.91) on the pre-metrics survey and 2.11 (1.05) on the post-metrics survey (*P* = .53).

## DISCUSSION

Given that all residents in this study chose to view their metrics, our results suggest that residents acknowledge they will possibly encounter performance metrics after graduation and are interested in viewing their data. This could also reflect a curiosity to see personalized, objective feedback, even if they see the feedback as imperfect. Sharing these metrics with trainees resulted in an increased report of feeling prepared to receive productivity metrics after graduation. We suspect that the increased sense of preparedness that residents reported was entirely due to having had the experience of receiving these metrics since no additional coaching or education surrounding performance metrics was delivered to the residents.

Interestingly, our results also showed that residents reported a statistically significant decrease in how pressured they felt to change their productivity metrics after receiving them. Given the limited information gained from the survey, the exact reason for this decreased perceived pressure is unclear but could have been affected by 1) residents being advised that these metrics would not be used for their evaluations or 2) absence of clearly defined metric expectations from the residency’s leadership (although group-comparison metrics were available). It is also possible that residents perceived less pressure after viewing their metrics secondary to reported metrics revealing better performance than they had expected or simply removing an element of the previously unknown. These results indicate that exposure to metrics during training, without being attached to formal evaluations, may give residents an increase sense of feeling prepared without necessarily adding to the performance pressure they feel during training.

Additionally, our results indicate that exposure to a single set of performance metrics resulted in no significant change in residents’ desire to go into administrative leadership after graduation. Our data also show that although viewing their metrics did not change residents’ perceptions of metrics as motivating, actionable, or important, it is interesting to note that on average residents in this small, single-center sample found metrics to be motivating, actionable and important, with all pre- and post-metrics survey averages above the neutral response of 3 (neither agree nor disagree). Since graduates are likely to encounter performance metrics after residency, training and exposure to metrics during residency may be a valuable aspect of residency curricula. In this study, simple exposure to metrics, without additional training or attached consequences, resulted in senior residents reporting that they felt more prepared for postgraduate work while simultaneously decreasing perceived pressure on them to change their performance.

## LIMITATIONS

Limitations of this study include small sample size and being conducted in a single academic center. Additionally, only 73% of residents completed the post-metrics survey. This may have biased our results if those who chose to complete both surveys had different perceptions and motivations compared to those who chose to complete only the first survey. For example, it is possible that those who completed both surveys may have seen metrics as more valuable than those who only completed the first survey. The reasons for decreased completion from pre- to post-metrics survey may include survey fatigue or decreased resident interest in the study after their metrics had been delivered.

### Future Directions for Research

It would be helpful if future studies were to investigate the effect of attaching consequences to metric exposure, such as including them in formal evaluations. Additionally, comparing the performance and perceptions of graduates who received different types of metrics training during residency would be valuable. Lastly, further investigation of factors that motivate residents may uncover other ways to affect resident performance and better prepare them for graduation.

## CONCLUSION

The results of this study are important to advance the conversation on the use of productivity metrics in training and demonstrate that providing productivity metrics to trainees may help them feel more prepared for their careers without necessarily increasing the pressure they feel to change their performance during training. Additionally, residents may see performance metrics as motivating, important, and actionable when not directly tied to their personal evaluations.

## Figures and Tables

**Table 1:** Pre- and post-metrics survey questions* in a study designed to gauge emergency medicine residents’ interest in viewing their productivity metrics.

Psychological impact
Receiving performance metrics makes me feel pressured to change my performance (Pressured)
Receiving performance metrics motivates me to change my performance (Motivates)
Perspective
Performance metrics are an important measure of my work (Important)
Performance metrics are an actionable form of feedback (I am able to change my results) (Actionable)
Prepared
I feel prepared to receive ED performance metrics after graduation (Prepared)
Career plan
I am interested in exploring the operations and administration aspect of how an emergency department performs after graduation (Administrative leadership)

* Question labels are included in parentheses.

*ED*, emergency department.

**Table 2:** Summary of pre- and post-metrics survey answers using a 5-point Likert scale in a study designed to gauge residents’ perspectives on viewing their productivity metrics.

Question label	Pre-metrics mean (SD)	Post-metrics mean (SD)	*P*-value
Pressured	2.32 (0.80)	3.05 (0.71)	< .01*
Motivated	2.04 (0.68)	2.11 (0.81)	.77
Important	1.88 (0.73)	2.11 (0.81)	.34
Actionable	1.96 (0.61)	2.16 (0.60)	.29
Prepared	2.32 (0.95)	1.68 (0.58)	.01*
Administrative Leadership	1.92 (0.91)	2.11 (1.05)	.53

5-point Likert scale (1 = strongly agree, 5 = strongly disagree).

* Statistically significant values.

*SD*, standard deviation.
